# Frequent detection of *Streptococcus tigurinus* in the human oral microbial flora by a specific 16S rRNA gene real-time TaqMan PCR

**DOI:** 10.1186/s12866-014-0231-5

**Published:** 2014-08-24

**Authors:** Andrea Zbinden, Fatma Aras, Reinhard Zbinden, Forouhar Mouttet, Patrick R Schmidlin, Guido V Bloemberg, Nagihan Bostanci

**Affiliations:** 1 Institute of Medical Microbiology, University of Zurich, Zurich, Switzerland; 2 Clinic of Preventive Dentistry, Periodontology and Cariology, Center of Dental Medicine, University of Zurich, Zurich, Switzerland; 3 Institute of Oral Biology, Center of Dental Medicine, University of Zurich, Zurich, Switzerland; 4 Institute of Medical Virology, University of Zurich, Winterthurerstrasse 190, Zurich, CH-8057, Switzerland

**Keywords:** Streptococcus tigurinus, Specific RT TaqMan PCR, Periodontitis, Oral microbiome

## Abstract

**Background:**

Many bacteria causing systemic invasive infections originate from the oral cavity by entering the bloodstream. Recently, a novel pathogenic bacterium, *Streptococcus tigurinus*, was identified as causative agent of infective endocarditis, spondylodiscitis and meningitis. In this study, we sought to determine the prevalence of *S. tigurinus* in the human oral microbial flora and analyzed its association with periodontal disease or health.

**Results:**

We developed a diagnostic highly sensitive and specific real-time TaqMan PCR assay for detection of *S. tigurinus* in clinical samples, based on the 16S rRNA gene. We analyzed saliva samples and subgingival plaque samples of a periodontally healthy control group (n = 26) and a periodontitis group (n = 25). Overall, *S. tigurinus* was detected in 27 (53%) out of 51 patients. There is no significant difference of the frequency of *S. tigurinus* detection by RT-PCR in the saliva and dental plaque samples in the two groups: in the control group, 14 (54%) out of 26 individuals had *S. tigurinus* either in the saliva samples and/or in the plaque samples; and in the periodontitis group, 13 (52%) out of 25 patients had *S. tigurinus* in the mouth samples, respectively (*P* = 0.895). The consumption of nicotine was no determining factor.

**Conclusion:**

Although *S. tigurinus* was a frequently detected species of the human oral microbial flora, it was not associated with periodontal disease. Further investigations are required to determine whether *S. tigurinus* is a commensal or an opportunistic oral pathogen with a potential for development of invasive infections.

## Background

The human oral microbiome consists of a number of bacteria; most of them are non-pathogenic commensals or act as opportunistic pathogens [1]. Some oral bacteria are implicated in oral diseases such as dental caries and periodontitis, which are among the most common infections in humans. Periodontitis in particular represents an inflammatory disease that affects 15-47% of the world-wide population [2],[3] and contributes to the morbidity of other chronic diseases [4]. Although more than 700 species were shown to colonize the oral cavity [5], evidence suggests that only a few of them, such as *Aggregatibacter actinomycetemcomitans* or *Porphyromonas gingivalis*, are associated with the pathogenesis of periodontitis or systemic complications [6],[7].

In recent years, significant associations have been elucidated between periodontitis and other very common systemic diseases, including diabetes mellitus [8] and cardiovascular diseases [9]. This pathogenic association between the oral cavity and other parts of the human body is potentially triggered by oral bacteria entering the bloodstream, which increases the risk for invasive infections such as infective endocarditis [10]. *Streptococcus tigurinus* was recently identified as a novel pathogen associated with infective endocarditis, prosthetic joint infections or meningitis [11]-[13]. It has also been shown to be highly virulent in experimental animal models [14].

*S. tigurinus* belongs to the *Streptococcus mitis* group and is most closely related to *Streptococcus mitis*, *Streptococcus oralis*, *Streptococcus pneumoniae*, *Streptococcus pseudopneumoniae* and *Streptococcus infantis. S. tigurinus* forms α-hemolytic, smooth colonies with a diameter of 0.5 to 1 mm after incubation at 37°C for 24 h on sheep blood agar [11]. Because of the morphological resemblance to its most closely related species, accurate identification of *S. tigurinus* by conventional phenotypic methods is limited. Therefore, commercial test systems such as VITEK 2 (bioMérieux, Marcy L’Etoile, France) or matrix-assisted laser desorption ionization-time of flight mass spectrometry analyses are helpful for initial assignment to the *S. mitis* group, but genetic analyses are required for definitive assignment as *S. tigurinus*. Analysis of the 5′-end of the 16S rRNA gene allows accurate identification of *S. tigurinus* based on a significant sequence demarcation to the most closely related species [11].

To date, the oral cavity *per se* could not yet be identified as niche of *S. tigurinus*. In addition, no data exists, whether or not *S. tigurinus* is a frequent commensal of the human oral cavity. Therefore, a *S. tigurinus* specific real-time (RT) TaqMan PCR based on the 16S rRNA gene was developed to identify *S. tigurinus* directly in clinical oral samples. In this context, saliva and dental plaque samples from a non-periodontitis control group and periodontitis patients as a test group were investigated as we hypothesized that the prevalence of *S. tigurinus* may be influenced by periodontitis. In addition, the influence of smoking on the occurrence of *S. tigurinus* was assessed.

## Methods

### Study population

Human saliva samples and pooled plaque samples of two different groups, i.e., a non-periodontitis control group (n = 26; 18 females, mean age 27.7 years, range 16 to 58) and a periodontitis group (n = 25; 14 females, mean age 59.4 years, range 26 to 83) of patients of the Center of Dental Medicine, University of Zurich, Switzerland, were prospectively analyzed. This study was approved by the Ethics committee of the canton Zurich, Switzerland (reference number KEK-ZH-2012-0322) and was conducted according to the guidelines of the Declaration of Helsinki. Pregnant patients or patients under antibiotic therapy were excluded from the study. All patients gave their written informed consent for the study. Clinical data were retrieved from the patients’ medical and dental records. Smoking status was anamnestically registered.

### Periodontal health status

In order to assess the periodontal health status of the patients, a periodontal examination was performed using a pressure-sensitive probe (Hawe Click Probe, Kerr Hawe, Bioggio, Switzerland), which included measurement of probing pocket depth (PPD) at six sites around each tooth. The dichotomous measurement of bleeding on probing (BOP) and presence of plaque/calculus or overhanging restorations were also recorded. All recordings were made by one calibrated investigator.

Based on this clinical data set, the periodontal health status was assessed by the periodontal screening index (PSI). This index provides an overall expression of the health status of the periodontium by assessing the PPD and BOP [15].

In brief, the staging is as follows: grade 0: no pockets >3 mm and no bleeding, grade 1: no pockets >3 mm, but presence of bleeding, grade 2: no pockets >3 mm, presence of bleeding plus the presence of calculus and/or overhanging restorations, grade 3: pockets of 4–5 mm, grade 4: pockets ≥6 mm. The highest score of a subject determined the clinical diagnosis according to the definition of Cutress and co-workers [16]: scores 0, 1, and 2: “no periodontitis”; scores 3 and 4: “periodontitis”.

### Clinical sample collection

Saliva samples of each patient were obtained by paraffin stimulation for 5 min. In addition, one week after the periodontal charting, subgingival plaque samples were collected from the four deepest pockets in the periodontitis group and from the mesial sulcus of the first molars in the non-periodontitis control group by paper points and curette method as described earlier [17]. Four subgingival plaque samples were pooled together for each patient.

### Primer design and TaqMan hydrolysis probes

**Figure 1:**
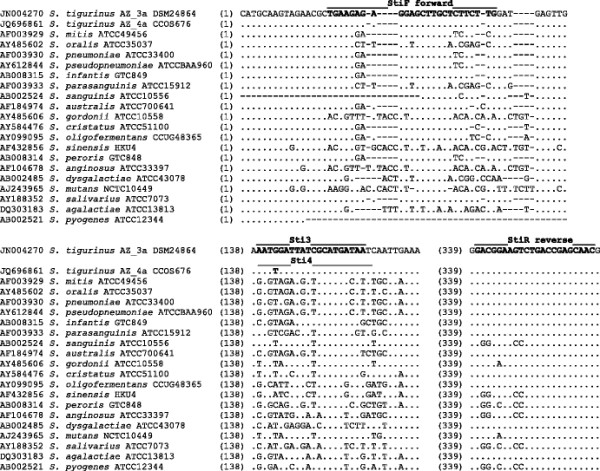
**Homology analysis of partial 16S rRNA gene sequences of*S*.*tigurinus*strains,*S*.*mitis*group species and more distantly related streptococci shows hypervariable regions.** Multiple alignment of the sequences was performed with the Clustal V program, sequence of the type strain *S. tigurinus* AZ_3a^T^ (CCOS 600^T^; DSM 24864^T^), is the reference sequence. The lines above the reference sequence depict the positions of the forward and reverse primers and the *S. tigurinus* specific TaqMan probes Sti3 (specific for *S. tigurinus* AZ_3a) and Sti4 (specific for *S. tigurinus* AZ_4a).

To establish a *S. tigurinus* specific RT TaqMan PCR, 16S rRNA gene sequences of *S. mitis* group species available from GenBank database and of *S. tigurinus* type strain AZ_3a^T^ (GenBank accession number JN004270) and *S. tigurinus* strain AZ_4a (JQ696861) were aligned using Clustal V program (Lasergene MegAlign software version 7, DNAstar, Madison, WI) (Figure 1). The sequence of *S. tigurinus* strain AZ_4a was included in the alignment as we observed a single nucleotide polymorphism at nucleotide position 150 at the 5′-end of the 16S rRNA gene. RT-PCR primers and TaqMan hydrolysis probes were chosen using PrimerExpress software version 3.0 (Life Technologies, Zug, Switzerland) following visual inspection of the aligned target sequences: forward primer StiF [5′-TGAAGAGAGGAGCTTGCTCTTCTTG-3′], reverse primer StiR [5′-GTTGCTCGGTCAGACTTCCGTC-3′], probe Sti3 [5′-6-FAM-AATGGATTATCGCATGATAA-MGB-3′, where FAM is 6-carboxyfluorescein and MGB is minor groove binder] and probe Sti4 [5′-NED-AATTGATTATCGCATGATAAT-MGB-3′, where NED is 2,7′,8′-benzo-5′-fluoro-2′,4,7-trichloro-5-carboxyfluorescein].

### DNA extraction and RT TaqMan PCR

DNA was extracted with an EZ1 DNA Tissue Kit (Qiagen, Hombrechtikon, Switzerland) following the manufacturer’s instructions. DNA extracts were eluted in 50 μl of PCR-grade water (Limulus amebocyte lysate [LAL] water; Lonza, Walkersville, MD). RT TaqMan PCR was performed on an Applied Biosystems 7500 fast instrument with 7500 System software (version 2.0.4). Each 25 μl mixture contained 12.5 μl of 2x PCR Mastermix (Roche Diagnostics, Rotkreuz, Switzerland), 2.5 μl of 10x exogenous internal positive-control primer and probe mix (VIC-labeled), 0.5 μl of 50x exogenous internal positive-control target DNA (both, Life Technologies), 0.25 μl of each primer (stock concentration, 30 μM), 0.5 μl of each probe (stock concentration, 5 μM), and 5.0 μl of DNA extract. The exogenous internal positive-control reagents were added to distinguish truly negative from falsely negative results due to PCR inhibition. PCR conditions were 2 min at 50°C and 10 min at 95°C, followed by 40 cycles of 15 s at 95°C and 60 s at 60°C. The positive-control plasmid pST3A containing a 435-bp segment of the 5′-end of the 16S rRNA gene (corresponding to positions 10 to 444 of the 16S rRNA gene of *S. tigurinus* AZ_3a^T^), containing the region as depicted in Figure 1, was constructed using *in silico* design and *de novo* synthesis and subcloning (Genscript, CA). The analytical sensitivity of the assay was determined by repeated testing of 10-fold dilutions of the plasmid positive control pST3A ranging from 5 × 10^5^ to 5 × 10^−1^ copies. PCR-grade water (LAL water) was used as a negative control. Sensitivity was evaluated by testing DNA extracts of *S. tigurinus* strains AZ_1 (CCOS 683, Culture Collection of Switzerland), AZ_2 (CCOS 675), AZ_3a^T^ (CCOS 600^T^; DSM 24864^T^, Deutsche Sammlung von Mikroorganismen und Zellkulturen GmbH, Braunschweig, Germany), AZ_4a (CCOS 676), AZ_6 (CCOS 681), AZ_7a (CCOS 677), AZ_8 (CCOS 678), AZ_10 (CCOS 679), AZ_11 (CCOS 682), AZ_12 (CCOS 680) and AZ_14 (CCOS 689); and of DNA extracts of 5 uncultured *S. tigurinus* (GenBank accession numbers JQ696868, JQ696870, JQ696871, JQ696872, JQ820471). Specificity was evaluated by testing DNA extracts of closely related streptococci, i.e., type strains of *S. pneumoniae* (DSM 20566^T^), *S. mitis* (DSM 12643^T^), *S. oralis* (DSM 20627^T^), *S. pseudopneumoniae* (CIP 108659^T^, Institut Pasteur, Paris, France) and *S. infantis* (CIP 105949^T^); and of clinical isolates of *Streptococcus gordonii*, *Streptococcus sanguinis*, *Streptococcus parasanguinis*, *Streptococcus salivarius*, *Streptococcus anginosus*, *Streptococcus mutans* and *Streptococcus dysgalactiae*. To further assess the assay specificity, amplification products from a sample tested positive with the *S. tigurinus* probes was sequenced and compared to known sequences using the NCBI BLAST tool and SmartGene software (SmartGene, Zug, Switzerland).

### Statistical analyses

The crosstab chi-square test of independence was performed by the IBMS PSS statistic software version 20. *P* < 0.05 was considered statistically significant.

## Results

### Development of a RT-PCR for the detection of *S. tigurinus*

A TaqMan-based RT-PCR for highly sensitive and specific detection of *S. tigurinus* in clinical samples was developed. A 288-bp fragment at the 5′-end of the 16S rRNA gene was selected, which allowed discrimination between *S. tigurinus* and the most closely related species within the *S. mitis* group (Figure 1). All *S. tigurinus* samples including *S. tigurinus* strain AZ_4a were detected due to the incorporation of two probes Sti3 and Sti4, respectively. Closely related species such as *S. pneumoniae*, *S. mitis*, *S. oralis*, *S. pseudopneumoniae* and *S. infantis* were not detected by the *S. tigurinus* specific RT-PCR, as well as other more distantly related species, i.e., *S. gordonii*, *S. sanguinis*, *S. parasanguinis*, *S. salivarius*, *S. anginosus*, *S. mutans* and *S. dysgalactiae*, showing the specificity of the assay.

Repeated testing of 10-fold serial dilutions of purified pST3A DNA consistently showed that the limit of detection for *S. tigurinus* was around 5 copies of the 16S rRNA gene using the Sti3 probe. In addition, specificity of the assay was supported by the lack of reactivity of the Sti4 probe with pST3A, which contains the 16S rRNA gene of *S. tigurinus* strain AZ_3a^T^. No amplification was detected for a template dilution of less than 5 copies and the negative control.

### Detection of *S. tigurinus* in the human oral cavity

In total, 51 saliva samples and 51 subgingival plaque samples obtained of 51 individuals were analyzed. In 22 (43%) out of 51 saliva samples and in 18 (35%) out of 51 plaque samples, *S. tigurinus* was detected by the specific RT-PCR, respectively. Overall, in 27 (53%) out of 51 individuals, *S. tigurinus* was detected in the saliva samples and/or in the plaque samples. In 13 (26%) individuals, *S. tigurinus* was detected both in the saliva and in the plaque samples. When comparing age groups <39 yr (n = 25), 40–65 yr (n = 16) and >65 yr (n = 10), no significant difference was observed for detection of *S. tigurinus* in the oral samples (*P* = 0.756).

Systemic comorbidities of patients were as follows: diabetes mellitus (n = 5), coronary heart disease (n = 3), rheumatoid arthritis (n = 1) and juvenile polyarthritis (n = 1); no immunosuppression was observed.

### Influence of periodontitis in the occurrence of *S. tigurinus*

Clinical diagnosis of periodontitis was based on the PSI. Individuals of the non-periodontitis control group (n = 26) had PSI grades <3 whereas patients of the periodontitis group (n = 25) had PSI grades 3 (n = 2) and 4 (n = 23). There is no significant difference of the frequency of *S. tigurinus* detection by RT-PCR in the saliva and dental plaque samples in the two groups: in the control group, 14 (54%) out of 26 individuals had *S. tigurinus* either in the saliva samples and/or in the plaque samples, and in the periodontitis group, 13 (52%) out of 25 patients had *S. tigurinus* in the mouth samples, respectively (*P* = 0.895) (Tables 1 and 2). Four (15%) out of 26 individuals of the non-periodontitis group and 9 (36%) out of 25 patients of the periodontitis group had *S. tigurinus* in both the saliva and the plaque samples, respectively (*P* = 0.091).

**Table 1:** **Frequency of** ***S*.** ***tigurinus*** **detected in the oral microbial flora of the periodontally healthy subjects (n = 26) by specific RT TaqMan PCR**

**Individual**	**Age, sex**	**Nicotine consumption**	**Detection of** ***S*.** ***tigurinus*** **in saliva sample by RT-PCR**	**Detection of** ***S*.** ***tigurinus*** **in subgingival plaque sample by RT-PCR**
1	23, f	Yes	Negative	Positive
2	23, f	Yes	Negative	Negative
3	18, f	No	Negative	Negative
4	18, f	No	Positive	Negative
5	22, f	Yes	Positive	Positive
6	16, f	No	Positive	Negative
7	23, f	No	Positive	Negative
8	18, f	Yes	Negative	Negative
9	39, f	Yes	Positive	Positive
10	16, f	Yes	Negative	Negative
11	26, f	No	Negative	Negative
12	26, m	No	Negative	Negative
13	24, f	No	Negative	Negative
14	48, m	No	Positive	Negative
15	31, m	Yes	Negative	Negative
16	53, m	No	Negative	Negative
17	24, f	No	Positive	Positive
18	26, f	No	Positive	Negative
19	33, m	No	Negative	Positive
20	58, m	No	Negative	Negative
21	25, m	No	Positive	Positive
22	23, m	Yes	Positive	Negative
23	34, f	No	Negative	Negative
24	25, f	No	Negative	Negative
25	24, f	No	Negative	Positive
26	25, f	No	Positive	Negative

**Table 2:** **Frequency of** ***S*.** ***tigurinus*** **detected in the oral microbial flora of the periodontitis group (n = 25) by specific RT TaqMan PCR**

**Patient**	**Age, sex**	**Nicotine consumption**	**Detection of** ***S*.** ***tigurinus*** **in saliva sample by RT-PCR**	**Detection of** ***S*.** ***tigurinus*** **in subgingival plaque sample by RT-PCR**
1	50, f	Yes	Positive	Negative
2	69, m	Yes	Negative	Negative
3	63, f	No	Negative	Negative
4	46, f	Yes	Negative	Positive
5	43, f	No	Negative	Negative
6	71, m	Yes	Negative	Positive
7	66, m	No	Negative	Negative
8	52, f	Yes	Positive	Positive
9	74, m	Yes	Positive	Positive
10	50, f	No	Positive	Positive
11	54, m	No	Negative	Negative
12	26, f	No	Positive	Positive
13	55, f	No	Negative	Negative
14	51, f	Yes	Negative	Negative
15	83, m	No	Positive	Positive
16	65, f	No	Positive	Negative
17	82, f	No	Positive	Positive
18	57, f	Yes	Positive	Positive
19	38, f	No	Positive	Positive
20	76, m	No	Negative	Negative
21	65, m	Yes	Negative	Negative
22	63, m	Yes	Negative	Negative
23	45, m	Yes	Positive	Positive
24	74, m	Yes	Negative	Negative
25	68, f	No	Negative	Negative

### Influence of nicotine consumption in the occurrence of *S. tigurinus*

In total, 20 out of 51 individuals had nicotine consumption, of which 11 had *S. tigurinus* detected in at least the saliva and/or plaque samples. This was not significant compared to individuals without nicotine consumption (31 out of 51, 16 with *S. tigurinus* detected in the oral samples), *P* = 0.813. In the periodontitis group, the number of patients with nicotine consumption and *S. tigurinus* detected in the oral samples (n = 7) did not differ significantly from the patients without nicotine consumption and *S. tigurinus* in the mouth (n = 6), *P* = 0.543, respectively. Similar results were observed in the non-periodontitis control group, 4 individuals with nicotine consumption and *S. tigurinus* detected in the oral samples were identified compared to 10 individuals without nicotine consumption but *S. tigurinus* detected in the mouth, *P* = 0.793.

## Discussion

Members of the microbial flora originating from the oral cavity may be involved in the pathogenesis of systemic infections [18]. Biofilm formation, complex mechanisms with other bacteria or underlying diseases might play a crucial role in the development of invasive infections. Regarding the pathogenesis of chronic periodontal diseases, complex host-bacterial interactions are responsible for the initiation of tissue destruction [19],[20]. Earlier studies have demonstrated that *S. mitis*, which is the closest related species to *S. tigurinus*, is a predominant early colonizing species of dental biofilms [21]. Although *S. mitis* is not a potent inducer of immune responses, it can antagonize the capacity of *A. actinomycetemcomitans* to stimulate IL-8 [22]. Interaction of *S. tigurinus* with *A. actinomycetemcomitans* (a key pathogen associated with aggressive form of periodontitis in younger individuals) might be of interest [23]. Since its recent identification [11],[12], it is not clear whether modifying factors are associated with the presence of *S. tigurinus* in the human oral microbiome and if its detection in the oral cavity has direct clinical implications in systemic diseases.

Our data shows that *S. tigurinus* is a frequent bacterium colonizing the human oral cavity in periodontal health and disease. In more than half of the individuals (53%) investigated *S. tigurinus* was detected by the *S. tigurinus* specific RT-PCR. Overall, the frequency of *S. tigurinus* in the saliva and plaque samples in patients with periodontitis did not differ significantly from individuals in the non-periodontitis control group. Both, individuals with or without nicotine consumption, had *S. tigurinus* in the saliva/plaque samples, independent of the individual’s age. However, it remains to be investigated how *S. tigurinus* interacts with other oral bacteria and if there might be a similar inhibitory effect.

Whole-genome analyses of *S. tigurinus* revealed the presence of several virulence factors such as fibronectin-binding protein or exfoliative toxin [24], which might differentiate this bacterium from other oral commensal organisms of the normal microbial flora. However, little is understood how exactly *S. tigurinus* causes various invasive diseases. An enhanced resistance to phagocytosis by macrophages of *S. tigurinus* was shown without induction of platelet aggregation [14].

Previous studies have shown that *S. tigurinus* is a frequent and aggressive pathogen causing infective endocarditis [11],[12],[14]. For patient management and guidance of appropriate therapy, accurate identification of the causative agent is of major importance. The *S. tigurinus* specific RT-PCR allows accurate discrimination between *S. tigurinus* and the most closely related species within the *S. mitis* group. In future, the *S. tigurinus* specific RT-PCR might be useful for direct application on clinical samples, e.g., heart valves, for timely identifications of the pathogen in a routine diagnostic laboratory.

The human oral microbiome is comprised of a bacterial diversity including different phyla, e.g., *Firmicutes*, *Actinobacteria*, *Proteobacteria*, *Bacteroidetes* and *Proteobacteria*[5],[25]. Viridans streptococci, e.g., *S. mitis*, are known to be the predominant bacterial species in the human oral cavity and were detected in various dental sites [5]. The present study is the first to show comparatively that *S. tigurinus* can be detected both in saliva and in subgingival plaque samples, however, it remains to be determined if the occurrence of *S. tigurinus* is site specific. It is not surprising that *S. tigurinus* can be found in saliva in higher frequency than individually selected subgingival sites, since saliva has representatively bacteria from different oral sites including the subgingival area. Saliva has been shown to be a suitable biological fluid, alternative to pooled subgingival plaque samples for detection of oral bacteria such as newly identified *Synergistetes*[26].

## Conclusions

We developed a diagnostic, highly sensitive and specific RT TaqMan PCR for direct detection of *S. tigurinus* in clinical samples. The data of the present study suggests for the first time that *S. tigurinus* represents a prevalent organism of the oral microbiome and that its occurrence is not increased by the presence of periodontal disease or smoking. However, prospective studies with larger populations are required to determine whether *S. tigurinus* is a commensal or an opportunistic oral pathogen with a potential for development of invasive infections.

## Competing interests

The authors declare that they have no competing interests.

## Authors’ contribution

AZ contributed to the overall study design, analysis of molecular data and drafting the manuscript. FA contributed to the acquisition of the clinical samples and parameters. RZ contributed to the overall study design and laboratory data. FM contributed to the statistical analysis. PRS contributed to the overall study design, acquisition of clinical samples and critical revision of the draft. GVB contributed to overall study design, development of molecular methods and critical revision of the draft. NB contributed to the overall study design, acquisition of clinical samples and data and drafting the manuscript. All authors read and approved the final manuscript.

## References

[B1] Marsh PD Are dental diseases examples of ecological catastrophes? Microbiology 2003 149 Pt 2 279 294 10.1099/mic.0.26082-0 12624191

[B2] Albandar JM Underestimation of periodontitis in NHANES surveys J Periodontol 2011 82 3 337 341 10.1902/jop.2011.100638 21214340

[B3] Konig J Holtfreter B Kocher T Periodontal health in Europe: future trends based on treatment needs and the provision of periodontal services–position paper 1 Eur J Dent Educ 2010 14 Suppl 1 4 24 10.1111/j.1600-0579.2010.00620.x 20415972

[B4] Marcenes W Kassebaum NJ Bernabe E Flaxman A Naghavi M Lopez A Murray CJ Global burden of oral conditions in 1990–2010: a systematic analysis J Dent Res 2013 92 7 592 597 10.1177/0022034513490168 23720570 PMC4484374

[B5] Aas JA Paster BJ Stokes LN Olsen I Dewhirst FE Defining the normal bacterial flora of the oral cavity J Clin Microbiol 2005 43 11 5721 5732 10.1128/JCM.43.11.5721-5732.2005 16272510 PMC1287824

[B6] Papapanou PN Behle JH Kebschull M Celenti R Wolf DL Handfield M Pavlidis P Demmer RT Subgingival bacterial colonization profiles correlate with gingival tissue gene expression BMC Microbiol 2009 9 221 10.1186/1471-2180-9-221 19835625 PMC2771036

[B7] Socransky SS Haffajee AD Implications of periodontal microbiology for the treatment of periodontal infections Compt Rendus Geosci 1994 18 S684 S685 688–693; quiz S714-687 8039206

[B8] Lalla E Papapanou PN Diabetes mellitus and periodontitis: a tale of two common interrelated diseases Nat Rev Endocrinol 2011 7 12 738 748 10.1038/nrendo.2011.106 21709707

[B9] Beck JD Offenbacher S Systemic effects of periodontitis: epidemiology of periodontal disease and cardiovascular disease J Periodontol 2005 76 11 Suppl 2089 2100 10.1902/jop.2005.76.11-S.2089 16277581

[B10] Spellerberg B, Brandt C: ***Streptococcus***. In *Manual of clinical microbiology. Volume 1.* 10th edition. Edited by Versalovic J, Carroll KC, Funke G, Jorgensen JH, Landry ML, Warnock DW. Washington, DC: ASM Press; 2011:331–349.

[B11] Zbinden A, Mueller NJ, Tarr PE, Sproer C, Keller PM, Bloemberg G: ***Streptococcus tigurinus*****sp. nov., isolated from blood of patients with endocarditis, meningitis and spondylodiscitis.** *Int J Syst Evol Microbiol* 2012, **62**(Pt 12):2941–2945. 10.1099/ijs.0.038299-0 22357776

[B12] Zbinden A, Mueller NJ, Tarr PE, Eich G, Schulthess B, Bahlmann AS, Keller PM, Bloemberg GV: ***Streptococcus tigurinus*****, a novel member of the*****Streptococcus mitis*****group, causes invasive infections.** *J Clin Microbiol* 2012, **50**(9):2969–2973. 10.1128/JCM.00849-12 PMC3421813 22760039

[B13] Zbinden A, Quiblier C, Hernandez D, Herzog K, Bodler P, Senn MM, Gizard Y, Schrenzel J, François P: **Characterization of*****Streptococcus tigurinus*****small-colony variants causing prosthetic joint infection by comparative whole-genome analyses.** *J Clin Microbiol* 2014, **52**(2):467–474. 10.1128/JCM.02801-13 PMC3911336 24478475

[B14] Veloso TR, Zbinden A, Andreoni F, Giddey M, Vouillamoz J, Moreillon P, Zinkernagel AS, Entenza JM: ***Streptococcus tigurinus*****is highly virulent in a rat model of experimental endocarditis.** *Int J Med Microbiol* 2013, **303**(8):498–504. 10.1016/j.ijmm.2013.06.006 23856340

[B15] Lo Frisco C Cutler R Bramson JB Periodontal screening and recording: perceptions and effects on practice J Am Dent Assoc 1993 124 7 226 229 231–232 8335792 10.14219/jada.archive.1993.0256

[B16] Cutress TW Ainamo J Sardo-Infirri J The community periodontal index of treatment needs (CPITN) procedure for population groups and individuals Int Dent J 1987 37 4 222 233 3481626

[B17] Belibasakis GN Schmidlin PR Sahrmann P Molecular microbiological evaluation of subgingival biofilm sampling by paper point and curette APMIS 2014 122 4 347 352 10.1111/apm.12151 23879704

[B18] Deshpande RG, Khan M, Genco CA: **Invasion strategies of the oral pathogen*****Porphyromonas gingivalis*****: implications for cardiovascular disease.** *Invasion Metastasis* 1998, **18**(2):57–69. 10.1159/000024499 10364686

[B19] Kinane DF Bartold PM Clinical relevance of the host responses of periodontitis Periodontol 2000 2007 43 278 293 10.1111/j.1600-0757.2006.00169.x 17214845

[B20] Hamedi M, Belibasakis GN, Cruchley AT, Rangarajan M, Curtis MA, Bostanci N: ***Porphyromonas gingivalis*****culture supernatants differentially regulate interleukin-1beta and interleukin-18 in human monocytic cells.** *Cytokine* 2009, **45**(2):99–104. 10.1016/j.cyto.2008.11.005 19091595

[B21] Li J Helmerhorst EJ Leone CW Troxler RF Yaskell T Haffajee AD Socransky SS Oppenheim FG Identification of early microbial colonizers in human dental biofilm J Appl Microbiol 2004 97 6 1311 1318 10.1111/j.1365-2672.2004.02420.x 15546422

[B22] Sliepen I Van Damme J Van Essche M Loozen G Quirynen M Teughels W Microbial interactions influence inflammatory host cell responses J Dent Res 2009 88 11 1026 1030 10.1177/0022034509347296 19828891

[B23] Ennibi OK, Benrachadi L, Bouziane A, Haubek D, Poulsen K: **The highly leukotoxic JP2 clone of*****Aggregatibacter actinomycetemcomitans*****in localized and generalized forms of aggressive periodontitis.** *Acta Odontol Scand* 2009, **70**(4):318–322. 10.3109/00016357.2011.642002 22251014

[B24] Gizard Y, Zbinden A, Schrenzel J, François P: **Whole-genome sequences of*****Streptococcus tigurinus*****type strain AZ_3a and*****S. tigurinus*****1366, a strain causing prosthetic joint infection.** *Genome Announc* 2013, **1**(2):e00210–e00212. 10.1128/genomeA.00210-12 PMC3642253 23640198

[B25] Dewhirst FE Chen T Izard J Paster BJ Tanner AC Yu WH Lakshmanan A Wade WG The human oral microbiome J Bacteriol 2010 192 19 5002 5017 10.1128/JB.00542-10 20656903 PMC2944498

[B26] Belibasakis GN, Ozturk VO, Emingil G, Bostanci N: ***Synergistetes*****cluster A in saliva is associated with periodontitis.** *J Periodontal Res* 2013, **48**(6):727–732. 10.1111/jre.12061 23441995

